# BLM helicase overexpressed in human gliomas contributes to diverse responses of human glioma cells to chemotherapy

**DOI:** 10.1038/s41420-023-01451-9

**Published:** 2023-05-11

**Authors:** Kamil Wojnicki, Agnieszka Kaczmarczyk, Bartosz Wojtas, Bozena Kaminska

**Affiliations:** 1 Laboratory of Molecular Neurobiology, Nencki Institute of Experimental Biology, Warsaw, Poland; 2 Laboratory of Sequencing, Nencki Institute of Experimental Biology, Warsaw, Poland

**Keywords:** Senescence, Cancer therapeutic resistance

## Abstract

Most of anti-tumour therapies eliminate neoplastic cells by introducing DNA damage which ultimately triggers cell death. These effects are counteracted by activated DNA repair pathways to sustain tumour proliferation capacity. RECQL helicases family, including BLM, participate in DNA damage and repair, and prevent the replication stress. Glioblastoma (GBM) is a common, malignant brain tumour that inevitably recurs despite surgical resection, radiotherapy, and chemotherapy with temozolomide (TMZ). Expression and functions of the BLM helicase in GBM therapy resistance have not been elucidated. We analysed expression and localisation of BLM in human gliomas and several glioma cell lines using TCGA datasets, immunostaining and Western blotting. BLM depleted human glioma cells were generated with CRISPR/Cas9 system. Effects of chemotherapeutics on cell proliferation, DNA damage and apoptosis were determined with flow cytometry, immunofluorescence, Western blotting and RNA sequencing. We found upregulated *BLM* mRNA levels in malignant gliomas, increased cytosolic localisation and poor survival of GBM patients with high *BLM* expression. BLM deficiency in LN18 and LN229 glioma cells resulted in profound transcriptomic alterations, reduced cell proliferation, and altered cell responses to chemotherapeutics. BLM-deficient glioma cells were resistant to the TMZ and PARP inhibitor treatment and underwent polyploidy or senescence depending on the TP53 activity. Our findings of high BLM expression in GBMs and its roles in responses to chemotherapeutics provide a rationale for targeting BLM helicase in brain tumours. BLM deficiency affects responses of glioma cells to chemotherapeutics targeting PARP1 dependent pathways.

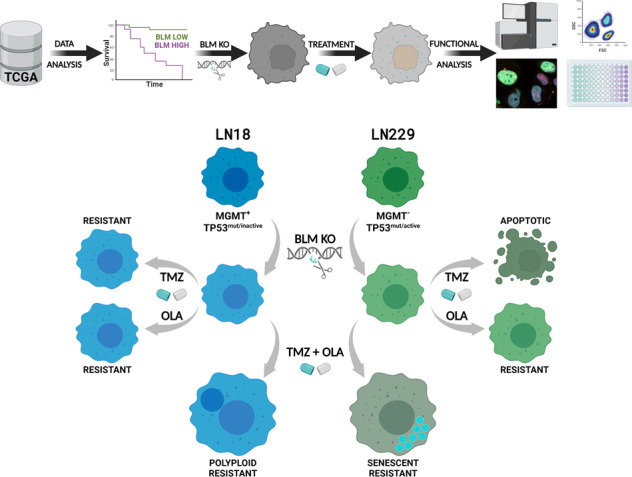

## Introduction

RecQ family helicases are involved in fundamental processes such as DNA repair, recombination, transcription, RNA decay and processing. They translocate along the DNA strands from 3’ to 5’ and separate the duplexes using the energy from ATP hydrolysis [1, 2]. The RecQ family consists of RECQL1, BLM, WRN, RECQL4, RECQL5 helicases, all of them playing an important role in genome maintenance and stability, and often referred as “guardians of the genome” [3, 4]. BLM (Bloom helicase) is involved in repair of DNA double-strand breaks by homologous recombination (HR) and participates in crucial steps of the process [5]. Germline mutations in *RECQL1*, *BLM, WRN* and *RECQL4* genes are manifested by genome instability and linked to the recessive autosomal syndromes and predisposition to cancer [6, 7]. Deleterious mutations in the *BLM* gene are associated with an increased risk of prostate, breast and colorectal cancers [8–10]. Disruption of *BLM* function in mice resulted in frequent sister chromatid exchange and developing cancer in heterozygous mutants or embryonic lethality of homozygous mice [11]. The expression of *BLM* is elevated in many cancers and the highest expression of *BLM* was reported in glioblastoma [12].

Glioblastoma (GBM) is the most common primary and aggressive brain tumour in adults [13]. Despite improved diagnosis, radical surgery, radio- and chemotherapy, GBM is essentially incurable due to diffusive growth and resistance to standard therapy. The median survival of GBM patients is 15 months [14, 15]. Chemotherapy with temozolomide (TMZ, a DNA alkylating drug) prolongs survival only by 2.5 months [16]. Over 50% of TMZ-treated patients poorly respond to TMZ due to expression of a repair enzyme, O^6^-methylguanine methyltransferase (MGMT), and/or dysregulation of DNA repair pathways in GBM cells [17]. Methylation of O^6^-guanine is a main mechanism of TMZ toxicity but the most frequent methylation occurs at N^7^-guanine (70%), which can be repaired via base excision repair (BER) [18]. Since 2005 FDA has not approved new drugs for GBM [19], there is an urgent need to discover and implement new therapeutic modalities.

Poly(ADP-Ribose) polymerase (PARP1) is involved in single-strand DNA break repair [20], therefore PARP1 inhibitors (PARPi) interfere with DNA repair [21] and might improve the TMZ toxicity. A clinical trial of TMZ with PARPi has been launched in GBM patients [22, 23].

Despite the importance of BLM in DNA repair and other processes, its involvement in glioma progression and responses of GBM cells to cytotoxic drugs have not been studied. We performed comprehensive analyses of BLM expression and functions in GBMs and demonstrated high levels of the *BLM* mRNA and protein in malignant tumours and glioma cell lines, along with abnormal cytoplasmic accumulation of BLM in GBMs. BLM deficiency in human LN18 and LN229 glioma cells affected cell growth, viability and responses to chemotherapeutics. We found that BLM deficient glioma cells become resistant to the combined TMZ and PARP1 inhibitor treatment and respond with polyploidy or cellular senescence. These processes are acquired mechanisms to escape from cell death. Our results point out that the BLM helicase is a plausible target in malignant gliomas to sensitise cells to chemotherapeutics. BLM expression could be one of the markers to predict responses to chemotherapeutics, particularly when TMZ is combined with PARP inhibitors, recently enroled in clinical trials.

## Results

### BLM helicase is overexpressed in human gliomas and glioma cells

**Fig. 1:**
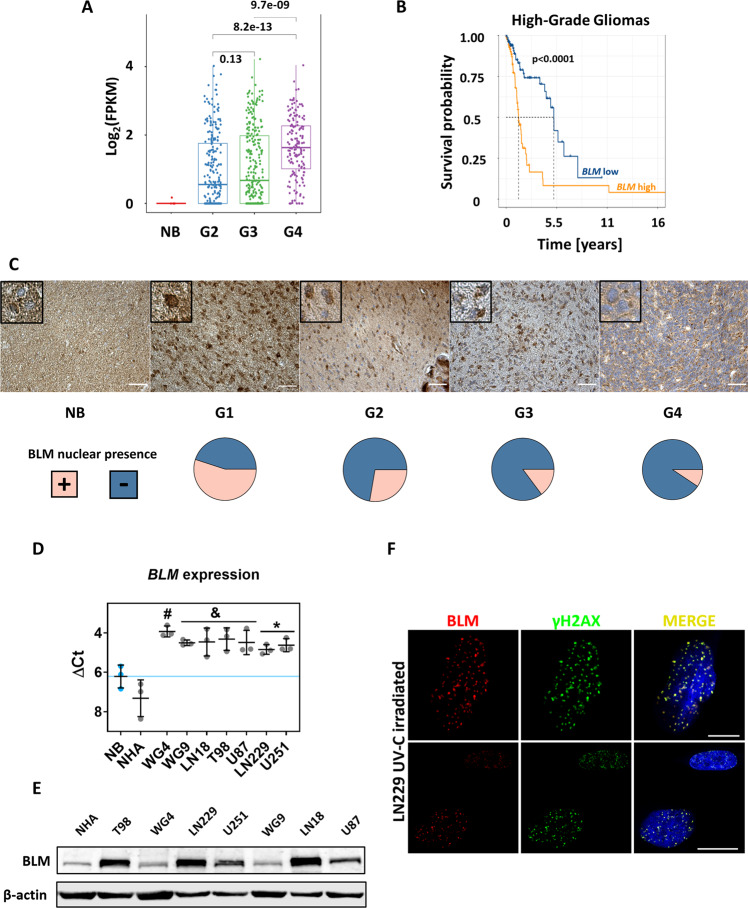
BLM is highly expressed in human glioma tumours and cell lines. **A** *BLM* mRNA expression in normal brain (NB), WHO grades 2, 3 glioma and WHO grade 4 glioblastoma in the Cancer Genome Atlas (TCGA) dataset. The expression is shown as log_2_ of FPKM (Fragments Per Kilobase of transcript per Million). The significance of *BLM* expression across glioma grades was determined using the Wilcoxon rank-sum non-parametric test. **B** Kaplan–Meier censored overall survival analysis from TCGA data of patients with high grade gliomas (WHO grades 3 and 4). The correlation between *BLM* expression and patient survival was assessed using the log-rank test and Kaplan–Meier estimators. **C** Representative images of BLM immunostaining in the glioma tissues using commercial tissue microarrays. Pie charts represent a number of specimens with the nuclear localisation of BLM across tumour grades. TMAs include a set of tissues from astrocytoma (*n* = 127), glioblastoma (*n* = 31), oligoastrocytoma (*n* = 7), oligodendroglioma (*n* = 9), ependymoma (*n* = 11), ganglioglioma (*n* = 1) and gliosarcoma (*n* = 1), plus tumour adjacent and normal brain (NB) tissues (*n* = 8). Statistical significance of the nuclear BLM presence was determined by chi-squared test (*p*-value = 6.52 × 10^−5^). High grade gliomas were IDH WT. Scale bar: 50 µm. **D** Expression of *BLM* in human established glioma cell lines (T98, LN229, U251, LN18, U87), patient-derived primary cultures (WG4, WG9), normal human astrocytes (NHA) and normal human brain (NB). RT-qPCR data are shown as delta Ct values relative to *GAPDH* expression. Mean delta Ct value of NB is indicated by blue solid line. Statistical analysis was performed using one-way ANOVA with Dunnett’s post-hoc test to normal brain (NB) (*p < 0.05, ^&^*p* < 0.01, ^#^*p* < 0.001), mean ± SD, *n* = 3. **E** Immunoblot showing BLM levels in various glioma cells and NHA. β-ACTIN was used as a loading control. **F** Representative images showing the immunofluorescent staining of BLM and γH2AX in LN229 cells irradiated with 30 J/m^2^ UV-C light. Scale bar: 10 µm.

Inspired by findings that BLM is overexpressed in many tumours, we analysed the *BLM* expression across the glioma patients using the transcriptomic dataset from the Cancer Genome Atlas (TCGA). We found elevated expression of *BLM* in gliomas of WHO grades 1–4, with the highest median expression of *BLM* in WHO grade 4 GBMs. Expression of *BLM* differed significantly between WHO grades 2 and 3 (Fig. 1A). The elevated expression of *BLM* in malignant gliomas correlated with the poor survival of patients (*p* < 0.0001). The median survival in high- and low-*BLM* expressing glioma patients was ~500 and 1950 days, respectively (Fig. 1B). Human glioma tissue arrays were employed to verify the BLM levels in gliomas of different WHO grades. In most cases, strong BLM positivity was present in the tumour tissue whereas BLM staining in the normal brains was barely detectable (Fig. 1C). In control brains and low grade gliomas most of the BLM staining was nuclear whereas both nuclear and cytoplasmic localisation of BLM was detected in malignant gliomas. Quantification of the staining results revealed the negative correlation between nuclear BLM positivity and glioma grades (Fig. 1C, pie charts).

We determined the BLM mRNA and protein levels in seven human glioma cell lines: established T98, LN229, U251, LN18, U87 cell lines and patient-derived primary WG4, WG9 cell cultures; normal human astrocytes (NHA) and normal brain (NB) served as a non-malignant control. BLM was uniformly significantly overexpressed in malignant cells in comparison to NB (Fig. 1D), with the highest median expression noted in WG4 cells. While BLM protein levels differed between the glioma cells (Fig. 1E), the highest level of BLM protein was detected in LN18 and LN229 cells. Therefore these cell lines were employed for further experiments. BLM levels were augmented when LN229 cells were exposed to the UV-C light to introduce double-strand breaks (DSB) into DNA, and immunofluorescence studies showed that BLM is co-localised with γ-H2AX, a marker of DSB (Fig. 1F). These results show overexpression of BLM in human gliomas and cells, and accumulation of the protein when DNA damage is induced.

### Identification of BLM related gene expression networks in glioma cells

**Fig. 2:**
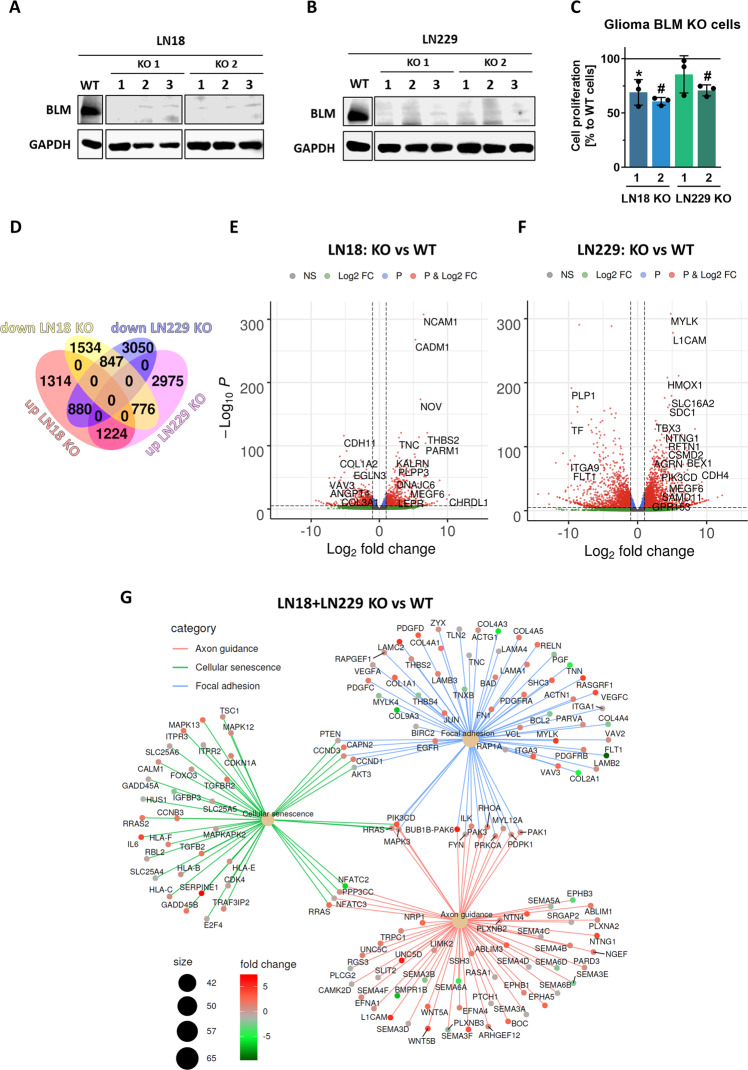
Transcriptomic changes in BLM-depleted glioma cells reveal affected gene networks. Immunoblots showing BLM levels in two (**A**) LN18 and (**B**) LN229 clones with BLM knockout (BLM KO) developed using CRISPR/Cas9 method, in three consecutive passages. Parental (wild type, WT) cells were used as a control. **C** Cell proliferation of LN18 and LN229 BLM KO glioma cells measured using BrdU assay. WT cell proliferation was set to 100% and is represented by a solid line. Statistical analysis was performed using one sample t-test (*p < 0.05, ^#^*p* < 0.001), mean ± SD, *n* = 3 in 5 replicates. **D** Venn diagram representing a number of commonly down- and upregulated genes LN18 and LN229 BLM KO cells. Volcano plots representing down- and upregulated genes in (**E**) LN18 and (**F**) LN229 BLM KO cells versus appropriate controls (log_2_ fold change < 0 and log_2_ fold change > 0, respectively, and FDR-adjusted *p* < 0.05). **G** Kyoto Encyclopedia of Genes and Genomes (KEGG) analysis of selected pathways commonly deregulated in LN18 and LN229 BLM KO cells when compared to WT cells, presented in a cnetplot gene network analysis.

We used the CRISPR/Cas9 genome editing to generate BLM depleted human LN18 and LN229 glioma cells. Genome editing efficacy was verified by Western blotting (Fig. 2A, B) and ultra-deep sequencing resulting in the selection of two clones/cell line. Proliferation of BLM-deficient (BLM KO) cells was significantly reduced: by 35% in LN18 BLM KO cells and 25% in LN229 BLM KO cells in comparison to respective wild type (WT) controls (Fig. 2C). As two independent clones showed similar responses, one clone/cell line was used for further experiments. BLM KO and WT glioma cells were subjected to RNA sequencing and differentially expressed genes (DEG) were identified. A Venn diagram (Fig. 2D) and volcano plots (Fig. 2E, F) show a large number of genes significantly up- and downregulated in the BLM KO cells in comparison to WT cells. In LN229 BLM KO cells 2975 genes were upregulated and 3050 genes downregulated. In LN18 BLM KO cells less genes were significantly changed: 1314 genes were up- and 1534 genes downregulated. Notably, 1224 up- and 847 downregulated genes were similarly regulated in both BLM KO cell lines indicating a common genetic network. We analysed DEGs using Kyoto Encyclopedia of Genes and Genomes (KEGG) and found numerous pathways and processes modified in BLM KO cells. The selected upregulated KEGG pathways (cellular senescence, focal adhesion and axon guidance) identified in a cneplot gene network analysis are presented (Fig. 2G).

### BLM deficiency affects the cytotoxic effects of the drug combination

**Fig. 3:**
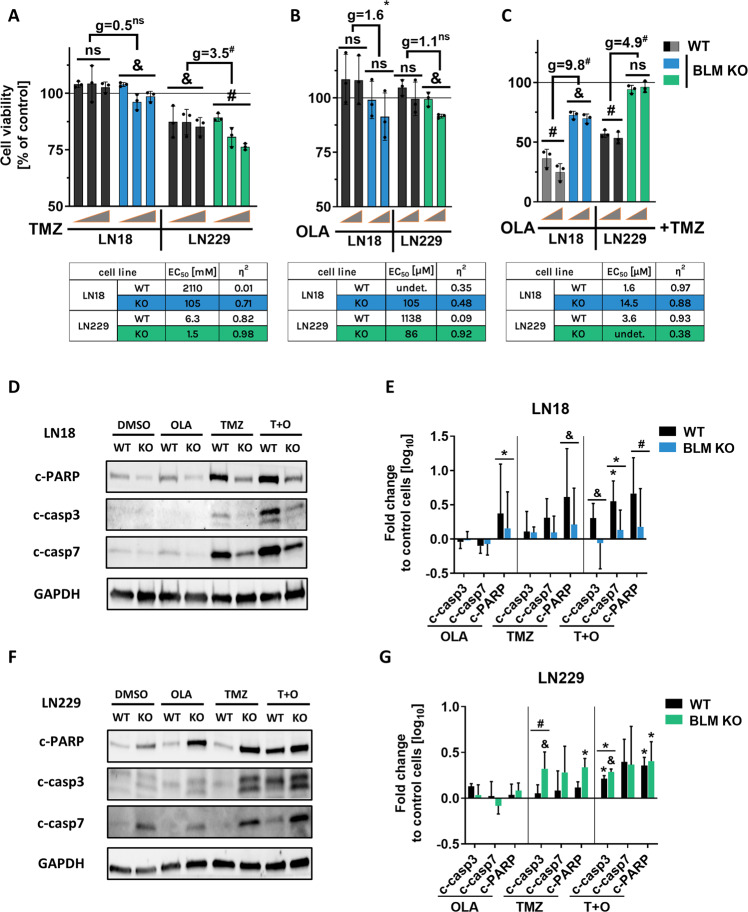
BLM deficiency in LN18 cells affects cell responses to the combined TMZ and PARP-1 inhibitors treatment. Viability of WT or BLM KO LN18 and LN229 cells after (**A**) temozolomide (TMZ), (**B**) olaparib (OLA) or (**C**) the combined TMZ + OLA treatments for 72 h. Cell viability of control cells set as 100% is represented by a black solid line. Grey triangles represent increasing doses of TMZ (50, 250 and 500 µM) and (**B**, **C**) OLA (1 and 5 µM) with 500 µM TMZ (**C**). EC_50_ values were calculated from linear regression from a dose-response relationship. Statistical analysis was performed using linear contrast ANOVA analysis (*p < 0.05, ^&^*p* < 0.01, ^#^*p* < 0.001), mean ± SD, *n* = 3. Hedge’s ‘g’ stands for effect size. Representative immunoblots detecting the apoptosis markers: cleaved PARP, cleaved caspase3 and cleaved caspase7 (c-PARP, c-casp3, c-casp7, respectively) of BLM KO and WT (**D**) LN18 and (**E**) LN229 cells after treatments with 500 µM TMZ, 1 µM OLA or 500 µM + 1 µM T + O. DMSO (0.5%) were added to control cells. GAPDH was used as a loading control. Densitometry of immunoblots showing levels of apoptotic proteins in BLM KO and WT (**F**) LN18 and (**G**) LN229 cells. GAPDH was used as a loading control. Statistical significance was determined by one-way ANOVA on logarithmic raw data followed by Dunnett’s post hoc test in comparison to untreated control cells (characters above the bars) or between the BLM KO and WT groups (characters above the lines), (*p < 0.05, ^&^*p* < 0.01, ^#^*p* < 0.001), *n* = 3, ±SD.

The gold standard treatment for GBM patients is maximal resection followed by temozolomide (TMZ) administration and radiotherapy [16]. Combining PARP inhibitors with homologous recombination deficiency (exemplified by BRCA1/2 deficiency) facilitates killing malignant cells due to synthetic lethality [21]. Recent clinical trials for GBM attempt to increase cytotoxicity by combining TMZ with PARP1 inhibitors such as olaparib (OLA) [22, 23]. We investigated if BLM deficiency would affect sensitivity of human glioma cells to chemotherapeutics, thus we treated LN18 and LN229 BLM WT and KO cells with TMZ (Fig. 3A), OLA (Fig. 3B) or with TMZ and OLA combination (Fig. 3C). We assessed the dose-response relationship towards TMZ (Fig. 3A) using MTT viability test and noticed that TMZ at the used doses had no impact on LN18 WT cells (EC_50_ = 2110 mM). LN18 BLM KO cells responded to the treatment (EC_50_ = 105 mM), however the calculated effect (g = 0.5) between the WT and KO groups was not significant. In contrast, we found significant dose dependency in LN229 BLM KO and WT cells after TMZ (Fig. 3A), and calculated EC_50_ values indicating 4x increased vulnerability towards TMZ (EC_50(WT)_ = 6.3 mM vs EC_50(KO)_ = 1.5 mM) with strong significant effect between the both groups (g = 3.5). We evaluated the sensitivity to OLA in aforementioned cells and we have not observed the dose-dependency in most cases (Fig. 3B). Nevertheless, we found out significantly increased vulnerability to OLA when compared LN18 WT and KO groups (EC_50(WT)_ = undet. vs EC_50(KO)_ = 105 μM, g = 1.6). Interestingly, when TMZ was combined with OLA, the response of glioma cells changed significantly. Both BLM KO cells became more resistant to the treatment, when compared to WT group (Fig. 3C). EC_50_ values for LN18 cells increased almost 10x (EC_50(WT)_ = 1.6 μM vs EC_50(KO)_ = 14.5 μM) whereas LN229 showed similar response (EC_50(WT)_ = 3.6 μM vs EC_50(KO)_ = undet). Strikingly, the effect size between BLM WT and KO for LN18 cells was much higher (g = 9.8) in contrast LN229 cells (g = 4.9), displaying stronger response to the drug combination.

These differences in responses of WT and BLM KO glioma cells to TMZ + OLA were corroborated at the levels of apoptosis markers: cleaved PARP [c-PARP], cleaved caspase 3 [c-casp3] and cleaved caspase 7 [c-casp7]. In LN18 cells, higher levels of those markers after TMZ or T + O treatment were detected in WT cells (Fig. 3D, E; Supplementary Fig. S2) suggesting a lower sensitivity of BLM KO cells. In LN229 cells the level of apoptotic markers in BLM KO increased after TMZ, and to a lesser extent, after the T + O treatment (Fig. 3E, F; Supplementary Fig. S2).

### The impact of PARP- and non-PARP inhibitors on BLM WT and KO glioma cell viability

**Fig. 4:**
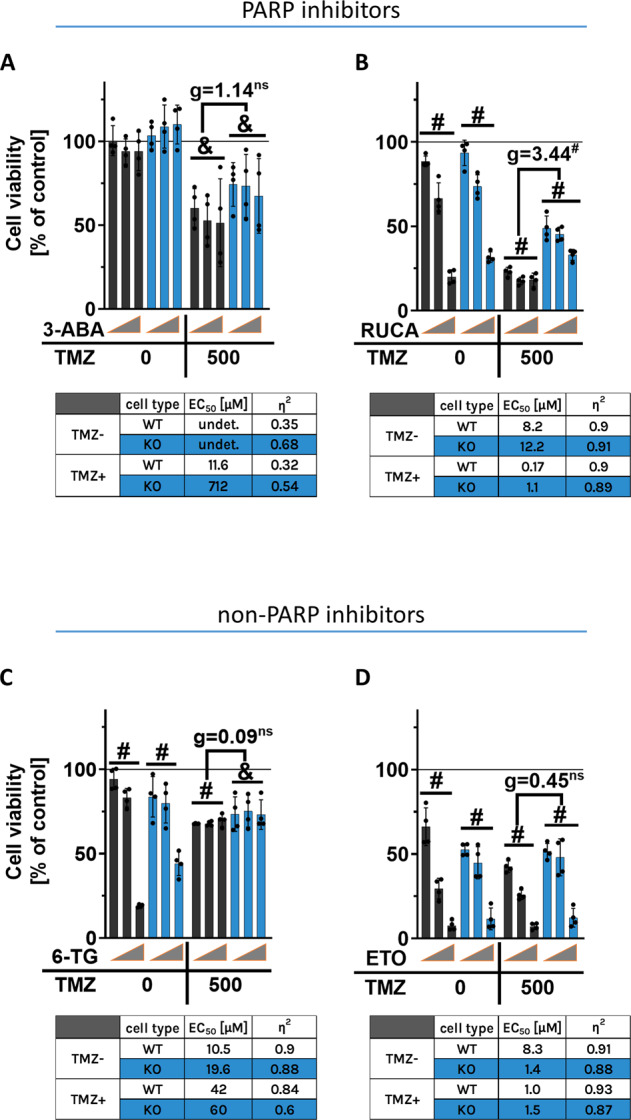
Effects of combining PARP or non-PARP inhibitors with TMZ on LN18 cells. Viability of LN18 WT and BLM KO cells after the treatment for 72 h with TMZ and PARP-1 inhibitors: (**A**) 3-aminobenzamide (3-ABA) and (**B**) rucaparib (RUCA), and non-PARP1 inhibitors treatments: (**C**) thioguanine (TG) and (**D**) etoposide (ETO) determined with a MTT cell metabolism test. Untreated control cells are indicated by a black solid line. Grey triangles represent increasing doses of compounds: 1, 5 and 25 µM, with constant 500 µM dose of TMZ. EC_50_ values were calculated from linear regression from dose-response relationship. Statistical significance was performed using linear contrast ANOVA analysis (^&^*p* < 0.01, ^#^*p* < 0.001), mean ± SD, *n* = 4. Hedge’s ‘g’ stands for effect size.

To decipher, whether the observed resistance is restricted to PARP-1 inhibitors, we tested cytotoxicity of TMZ in combination with other PARP-1 and non-PARP-1 inhibitors at given concentrations: 1, 5 and 25 μM (Fig. 4A–D) using MTT test. LN18 BLM KO cells were also resistant to the treatment of TMZ with other PARPi: 3-aminobenzamide (3-ABA, EC_50(WT)_ = 11.6 μM vs EC_50(KO)_ = 712 μM) (Fig. 4A) and rucaparib (RUCA, EC_50(WT)_ = 0.17 μM vs EC_50(KO)_ = 1.1 μM) (Fig. 4B) indicated by higher EC_50_ values in BLM KO groups when compared to WT cells. Moreover, the increasing ‘g’ values presented more potent action of RUCA towards glioma cells. LN18 BLM KO and WT cells co-treated with TMZ and non-PARPi: thioguanine (TG, EC_50(WT)_ = 42 μM vs EC_50(KO)_ = 60 μM) (Fig. 4C) or etoposide (ETO, EC_50(WT)_ = 1.0 μM vs EC_50(KO)_ = 1.5 μM) (Fig. 4D) showed similar levels of cell death and similar EC_50_ values, displaying no significant relationship after non-PARP inhibitors treatment. Notably, the Hedge’s ‘g’ emphasised significant differences only in TMZ with PARPi groups. These results demonstrate that BLM deficiency affects responses of glioma cells towards the clinically used chemotherapeutics targeting PARP-1 dependent pathways.

### Transcriptomic changes induced by chemotherapy in BLM-deficient cells define cell growth and senescence as targeted processes

**Fig. 5:**
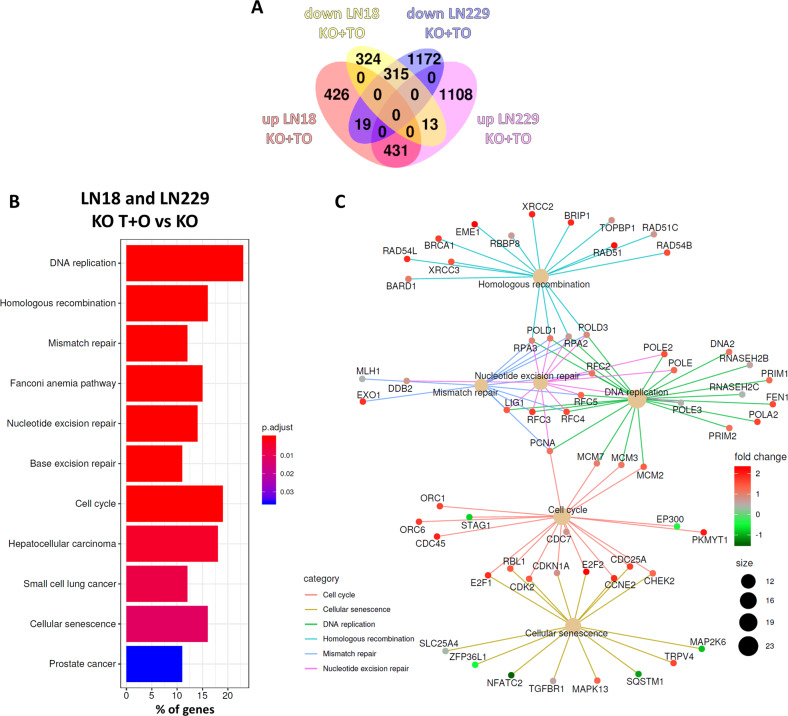
Identification of gene networks induced in BLM KO glioma cells by TMZ + OLA treatment. **A** Venn diagram shows a number of significantly (FDR-corrected *p* < 0.05) down- and upregulated genes in LN18 and LN229 BLM KO cells after the T + O treatment compared to controls. **B** KEGG analysis of differentially expressed genes displays commonly deregulated pathways in T + O-treated LN18 and LN229 BLM KO cells compared to controls. **C** Predominantly affected KEGG pathways were revealed by a cnetplot gene network analysis.

Studying global gene expression patterns and dissecting the enriched signalling affected by the treatment is a common way to estimate a role of a targeted protein. We performed RNA sequencing and computational analysis in search for specific processes affected by the BLM knockout. The analysis of transcriptomic changes in WT and BLM KO cells untreated or treated with the combination of T + O revealed a set of common genes upregulated (431) and downregulated (315) in drug-treated BLM KO glioma cells (Fig. 5A). KEGG analysis of DEGs showed many dysregulated functional pathways, when compared to untreated BLM KO glioma cells (Fig. 5B). The selected KEGG pathways detected in a cneplot gene network analysis are shown (Fig. 5C). Many genes from those pathways were upregulated in T + O-treated BLM KO cells, which indicates that the treatment stimulates processes related to the cell cycle regulation, DNA repair and senescence.

### BLM deficiency alters the cell cycle in glioma cells treated with TMZ and OLA

**Fig. 6:**
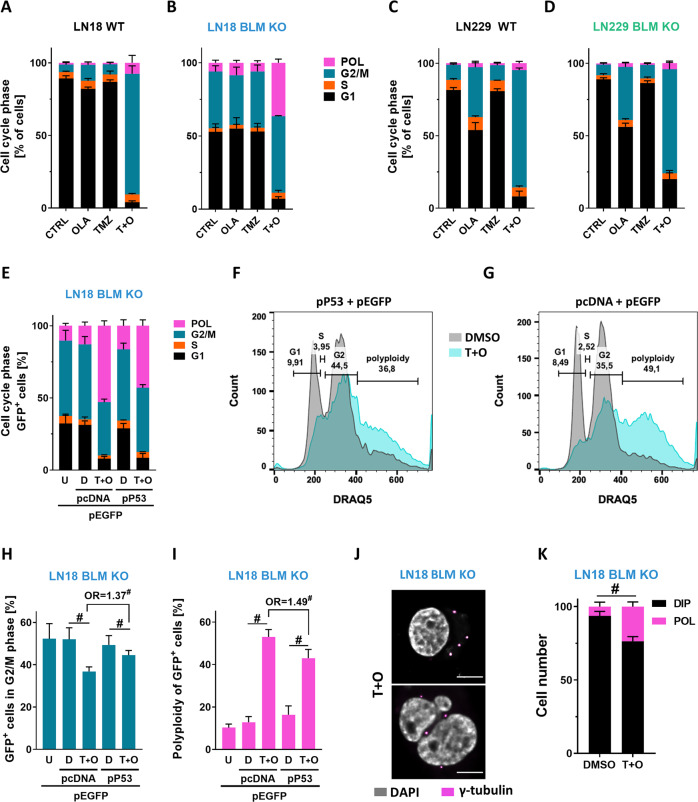
TMZ + OLA treatment leads to the cell cycle arrest in BLM KO glioma cells. Percentages of cells in phases of the cell cycle and polyploid states in cells: (**A**) LN18 WT, (**B**) LN18 BLM KO, (**C**) LN229 WT and (**D**) LN229 BLM KO glioma cells after the treatments as above. Cells were stained with propidium iodide and subjected to flow cytometry analysis, ≥10,000 events/sample, *n* = 3, mean ± SD. **E**–**I** Restoration of active p53 partly reduces polyploidy induced by the treatments. **E** Quantification of the cell cycle and ploidy in LN18 BLM KO cells co-transfected with control (pcDNA) or wt p53 (pP53) and EGFP (pEGFP) plasmids and subjected to the T + O treatment 24 h after transfection. Cells were stained with DRAQ5 dye and analysed using flow cytometry, *n* = 5, ≥10,000 GFP^+^ events/sample, mean ± SD. The pEGFP expressing cells (U) served as controls. The representative histograms of GFP-expressing LN18 BLM KO cells transfected with (**F**) pP53 or (**G**) pcDNA and treated with T + O are shown. Quantification of (**H**) G2/M and (**I**) polyploidy in LN18 BLM KO cells transfected with a given plasmid and treated with T + O. Statistical analysis was performed using chi-square test versus the controls (^#^*p* < 0.001) or between pcDNA and p53 group (^#^*p* < 0.001), *n* = 5, ≥10,000 GFP^+^ events/sample, mean ± SD. OR stands for odds ratio. OR = 1.37 CI95(1.33;1.40), OR = 1.49 CI95(1.46;1.53). **J** Representative staining for γ-tubulin (a centrosome marker) in the LN18 BLM KO cell after T + O. The pink dots depict the γ-tubulin foci. Nuclei were visualised using DAPI staining. Scale bars: 10 µm. **K** Quantification of γ-tubulin foci in LN18 BLM KO cells after T + O. Cells with 2 foci were considered as diploid (DIP), and with 4 or more, as polyploid (POL). Statistical analysis was performed using chi-square test (^#^*p* < 0.001), *n* = 3, 100 cells, mean ± SD.

The analysis of the cell cycle showed that under basal conditions a large fraction of LN18 BLM KO cells (but not LN229 cells) was in the G2/M phase (38%) in contrast to 5% of LN18 WT cells (Fig. 6A–D). The T + O treatment induced the cycle arrest in the G2/M phase in both LN18 and LN229 cells (Fig. 6A–D) but the strongest effects was in LN18 BLM KO cells (Fig. 6C). In LN18 BLM KO cells TMZ or OLA alone did not induce the cell cycle arrest but T + O resulted in significant polyploidisation in these cells (Fig. 6B). LN229 BLM KO and WT cells underwent the G2/M arrest after T + O and there was no induction of polyploid cells (Fig. 6D).

LN18 cells have inactive TP53 while TP53 remains partly active in LN229 cells [24]. To elucidate if p53 activity may influence polyploidisation, we co-transfected LN18 BLM KO cells with a plasmid carrying a functional TP53 (pP53) or the control plasmid (pcDNA) and a plasmid coding for enhanced green fluorescence protein EGFP (pEGFP). After the T + O treatment GFP expressing cells were analysed by flow cytometry (Fig. 6E and Supplementary Fig. S1 for a gating strategy). LN18 BLM KO cells transfected with pP53 showed reduced polyploidy after the treatment (Fig. 6F, G). Quantification of the cell cycle distribution showed the significant increase of the G2/M cells (Fig. 6H–J) and decrease of polyploid cells (Fig. 6I), with the odds ratio (OR) = 1.49. To confirm these results, we visualised centrosomes, the major microtubule organising centre of animal cells [25] by γ-tubulin immunostaining in BLM KO cells treated with T + O (Fig. 6J). Quantification of γ-tubulin signals showed increases of cells with more centromers (Fig. 6K). Altogether, the results show that the co-existence of BLM and TP53 deficiency in glioma cells reduces cytotoxicity after the TMZ + OLA treatment and results in polyploidy of glioma cells.

### T + O treatment evokes cellular senescence in LN229 BLM KO cells

**Fig. 7:**
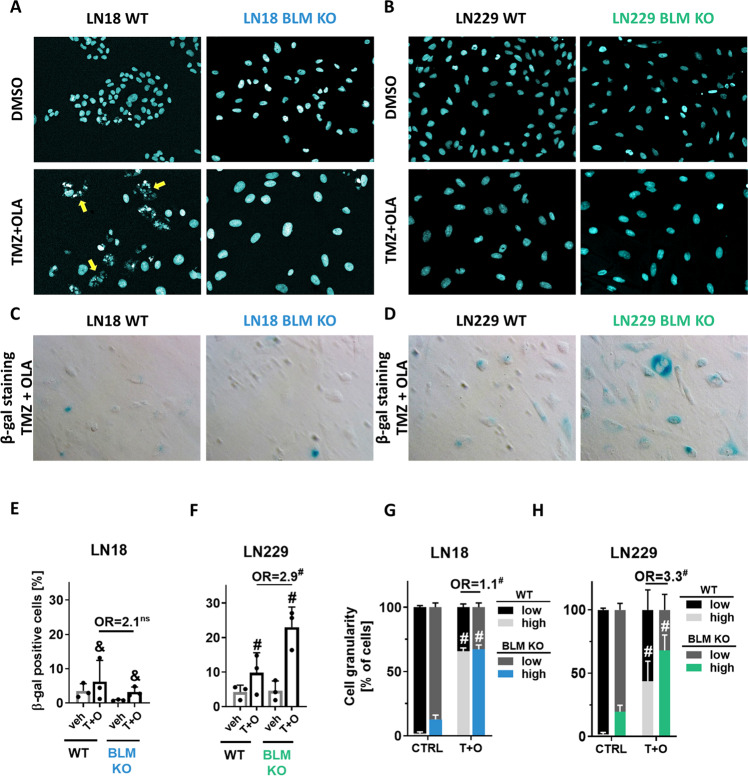
TMZ + OLA treatment evokes cellular senescence in LN229 BLM KO cells. Representative images of nuclei of BLM KO and WT (**A**) LN18 and (**B**) LN229 cells treated with 500 µM TMZ and 1 µM OLA (T + O); DMSO served as a control. Yellow arrows indicate cells with apoptotic, fragmented nuclei. Nuclei were visualised using DAPI staining. Representative images of β-galactosidase (β-gal) staining of (**C**) LN18 and (**D**) LN229 cells after T + O. Blue colour indicates cells with increased β-gal activity. Quantification of β-gal positive cells amongst T + O-treated (**E**) LN18 and (**F**) LN229 cells. Statistical analysis was performed using chi-square test in comparison of treated to control cells (above the bars, ^&^*p* < 0.01, ^#^*p* < 0.001) or between WT and BLM KO cells (above the line, ^#^*p* < 0.001), *n* = 3, in duplicates, mean ± SD. OR stands for odds ratio. OR = 2.1 CI95(1.1;3.9), OR = 2.9 CI95(2.0;4.2). Cell granularity of T + O-treated (**G**) LN18 and (**H**) LN229 cells determined by flow cytometry. Statistical analysis was performed using chi-square test in comparison of treated to control cells (CTRL) (above the bars, ^#^*p* < 0.001) or between the WT and BLM KO cells (above the lines, ^#^*p* < 0.001), *n* = 3, ≥10,000 events/sample, ±SD. OR stands for odds ratio. OR = 1.1 CI95(1.0;1.1), OR = 3.3 CI95(3.2;3.4).

Transcriptomic changes in BLM KO cells show the enrichment of differentially expressed genes related to a cellular senescence. Light microscopy showed that LN18 BLM KO glioma cells exposed to T + O have the increased size and flattened cell body while the fragmented nuclei indicating apoptosis were found only in LN18 WT cells. A number of cells with the oversized nuclei increased in BLM deficient glioma cells after the T + O treatment (Fig. 7A, B). An enlarged size of cells is a marker of cellular senescence (Supplementary Fig. S3) [26]. We measured an activity of the β-galactosidase (β-gal), a lysosomal enzyme upregulated in senescent cells. While in LN18 WT and BLM KO cells β-gal positive cells were detected after T + O (Fig. 7C, E), a number of β-gal positive cells was high in control LN229 BLM KO cells and strongly increased after the treatment (Fig. 7D). Percentages of β-gal^+^ cells significantly increased only in T + O treated LN229 WT cells but even stronger increase was found in BLM KO cells (OR = 2.9) (Fig. 7E, F). Measurements of cell granularity (which is another marker of cellular senescence) by flow cytometry confirmed increased cellular granularity in LN18 cells independently of the BLM status (OR = 1.1) (Fig. 7G) and increases in percentages of highly granular LN229 BLM-deficient cells (OR = 3.3) after T + O (Fig. 7H). These results show that the T + O treatment induces cellular senescence mostly in BLM deficient cells helping those cells to escape cell death.

## Discussion

In this study we demonstrate that BLM, a member of REQL helicase family, is upregulated in malignant gliomas and impacts GBM patient survival. BLM deficiency in glioma cells changes their responses to chemotherapy and shifts their responses to apoptosis, polyploidy or cell senescence.

RecQ helicases participate in important cellular events including replication, transcription, recombination and DNA repair cooperating with many enzymes including PARP1 [4]. As BLM expression is upregulated in many tumours including GBMs, we sought to define the role of BLM helicase in human glioma cells and their vulnerability to clinically used chemotherapeutics. We show that mRNA and protein expression of BLM is elevated in malignant gliomas, and high levels of *BLM* mRNA correlate with poor survival of GBM patients. Interestingly, we reported for a first time that BLM is localised mostly in a cytoplasm of malignant gliomas (WHO grades 3 and 4) while in normal brain and low grade gliomas BLM was located primarily in the nuclei. Such different localisation indicates distinct functions of BLM in malignant tumours. The cytosolic BLM was found previously in breast cancers, where its abundance correlated with increased aggressiveness [27]. The aberrant overexpression of BLM in the cytoplasm in colorectal cancers was associated the CpG island promoter hypomethylation and increased DNA damage responses [28]. The *BLM* mutations resulting in alterations in the nuclear targeting signal (NLS) domain [29] or dysregulation of mechanisms responsible for nuclear targeting might be another reason for mis-localisation of BLM in glioma cells. Recently, new non-synonymous single nucleotide polymorphisms (nsSNP) in the *BLM* gene have been discovered. Twenty eight ‘stop gained’- and one ‘start lost’ nsSNPs mutations may result in the truncated form of BLM protein with the loss of nuclear signals and important domains responsible for nuclear localisation [30]. However, these predictions should be validated experimentally to ultimately decipher this phenomenon. The high *BLM* expression inversely correlated with survival of breast cancer [27] or cholangiocarcinoma patients [31].

BLM protein levels were elevated in all 7 tested glioma cell lines when compared to normal human astrocytes. Generating BLM KO cells was instrumental in dissecting its role in cellular processes. BLM deficiency in glioma LN18 and LN229 cells reduced their proliferation and LN18 BLM KO cells were significantly arrested in the G2/M phase. Transcriptomic patterns of LN18 BLM KO cells showed downregulation of mini-chromosome maintenance genes (*MCM2,3,4,5,6,7*), essential factors for transcription initiation; such changes were not detected in BLM KO LN229 cells. In both cell lines BLM-deficient cells had dysregulated functional pathways implicated in focal adhesion or tight junctions and displayed altered morphology. We found changes in genes associated with apoptosis and cellular senescence pathways that could modulate the response of BLM-deficient cells to chemotherapeutics.

Treatment with temozolomide, a DNA alkylating agent commonly used in GBM therapy, offers limited survival benefits, thus combining with PARP1 inhibitors is envisioned. PARPi were successfully used in breast and ovarian cancers with the BRCA1/2 deficiency [32]. The combination of TMZ and PARPi (olaparib) has been explored in a clinical trial of GBM. Increased chemosensitivity to TMZ in MSH6-inactivated gliomas and overcoming acquired chemoresistance caused by MMR deficiency have been reported [28]. We show slight sensitisation the BLM KO glioma cells to TMZ alone and distinct responses of BLM-deficient glioma cells to TMZ, OLA and T + O. PARP1 is one of the proteins cooperating with RECQL helicases in DNA repair and genome maintenance [33] and its inhibitors (such as OLA) are supposed to enhance cytotoxicity of DNA damaging drugs. TP53-deficient LN18 glioma cells do not responds to most chemotherapeutics, in contrast to LN229 cells with a partly active TP53 that respond relatively well. Moreover, LN18 cells have the DNA repair MGMT protein in contrast to LN229 cells [34]. Notably, the BLM deficiency strengthened the effect of TMZ in MGMT deficient cells, creating a new therapeutic opportunity for BLM inhibitors.

BLM KO cells exposed to T + O displayed the induction of polyploidy or cellular senescence, suggesting the induction of different mechanisms to escape cell death. In particular, LN229 BLM-deficient cells increased percentages of senescent, β-gal positive cells with high degree of granularity. This finding is important as the combination of TMZ and PARP1 inhibitors was tested in a clinical trial of GBM [22, 23] and is recommended in a basket trial (NCT01390571) [35]. The combined T + O treatment triggered cell death in WT LN18 and LN229 cells, however BLM knockout protected those cells from cell death. The stronger cytostatic/cytotoxic effects of the combination was evident in both glioma cell lines. BLM deficiency sensitised LN229 cells to TMZ and to lesser extent to olaparib. This observation is surprising as HR-deficient cells should display increased susceptibility to PARPi [21] and sensitisation of WT LN229 glioma cells towards TMZ by PARPi was described [36]. We do not have a plausible explanation why knockdown of BLM in glioma cells makes PARP inhibition ineffective. The results in the Figs. 3, 6 and 7 show that a lack of BLM blocked LN18 cells in the G2/M phase, inhibited T + O treatment induced apoptosis and shifted p53 proficient cells LN2229 cells to senescence. The data suggests that BLM is somehow important in inducing growth arrest and apoptotic cell death. BLM is required for PARP inhibitors to synergise with TMZ and induce cell death. These data suggests that BLM is somehow important in inducing growth arrest and apoptotic cell death. The insights to those variable effects are provided by the results of transcriptomic analysis. Transcriptomic changes induced in BLM KO cells by the T + O treatment included upregulation of genes implicated in DNA replication, DNA repair and cellular senescence pathways. LN18 BLM KO cells exposed to T + O were arrested in the cell cycle in the G2/M phase and underwent polyploidisation or cellular senescence. Both processes are considered as therapy-induced mechanisms of escaping cell death and senescent cells are frequently polyploid [37]. Tetraploid cells can arise due to the mitotic slippage from the prolonged mitotic arrest or cytokinesis failure [38]. As BLM participates in the correct chromosome segregation and resolving anaphase bridges [39], its deficiency in T + O-treated glioma cells may lead to the cytokinesis failure. Accordingly, LN18 BLM KO cells showed an increased percentage of cells with a β-galactosidase activity and polyploidy nuclei after the T + O treatment. The induction of death escape mechanisms was also due to TP53 deficiency, as restoration of the functional p53 in LN18 BLM KO cells reduced percentages of polyploid cells. The accumulation of polyploid LN229 cells after TMZ alone and TMZ combined with pamiparib (PARPi) was detected by other study [40]. Percentages of the cell cycle arrested cells and increased number of β-gal^+^ senescent cells after T + O treatment were significantly higher in LN229 WT cells than in LN229 BLM KO cells. T + O induced cellular senescence in LN229 BLM KO glioma cells as evidenced by the appearance of enlarged, β-gal^+^ cells and increased granularity. While inducing the permanent cell cycle arrest and cell senescence are rather negative events and represent the cell death evasion mechanisms, it could be targeted in cancer treatment using senolytic drugs [41]. As neoplastic cells can emerge from senescent cells after cessation of chemotherapy [42], the removal of senescent tumour cells is envisioned.

Altogether, our results show that a BLM helicase, which is upregulated in malignant gliomas, is a new target in tumour cells, which may pave way to the development of specific BLM inhibitors. BLM deficiency changes the way glioma cells respond to drugs and sensitises KO cells to therapeutics. Interestingly, certain BLM KO glioma cells are insensitive to the combination of TMZ and PARPi but not with other chemotherapeutics, it shows a need for better diagnostics and patient stratification. Our results show that BLM cooperates with PARP1 in post-therapy DNA repair and its lack make cells insensitive to PARPi. As the T + O treatment is already in the clinic, we propose that testing BLM expression and TP53 status could help to better predict a patient response to aforementioned treatment. With genetic alterations in the *BLM* gene reported in gliomas, manipulation of the BLM level could be additional strategy to improve the therapeutic option for patients with deadly brain tumours.

## Materials and methods

### Cell Culture and treatments

LN18, LN229, T98G (T98), U87-MG (U87) and U251 cell lines were purchased from the ATCC, USA, and cultured in DMEM medium (Dulbecco’s modified Eagle medium, Thermo Fisher Scientific, USA). Primary glioblastoma cell lines (WG4 and WG9) were derived as described [43] and cultured in DMEM/F12 GlutaMAX media (Thermo Fisher Scientific, USA). Normal human astrocytes (NHA, Lonza) were cultured in medium containing ABM^TM^ Basal Medium (CC-3187) and AGM^TM^ SingleQuots^TM^ Supplements (CC-4123) from Lonza.. All culture media were supplemented with 10% FBS (Gibco, USA), antibiotics (100 U/mL penicillin, 100 µg/mL streptomycin) and cultured in a humidified atmosphere of CO_2_/air (5%/95%) at 37 °C. Cells were treated with Temozolomide (Sigma, USA) alone or with combination with: Olaparib, 3-aminobenzamide, Rucaparib, Tioguanine or Etoposide. Aforementioned compounds were dissolved in DMSO. Irradiation of UV-C light was used with 30 J/m^2^ dose. For reagent specifications and catalogue numbers see the Supplementary Table S1.

### CRISPR/Cas9 mediated knockout of *BLM* in glioma cells

BLM knockout cells were generated using the custom designed CRISPRCLEAR™ Transfection Ready Kit (Applied Stem Cell, USA) for the human *BLM* gene. The kit included the validated gRNA in the expression vector targeting the *BLM* exon 2 and Cas9-Puro plasmid (Supplementary Table S1). Glioma cells were seeded at a density of 1.2 × 10^5^ cells and transfected with 0.75 µg of each plasmid using Lipofectamine 2000 (Invitrogen, USA) for 6 h, followed by puromycin selection (2 µg/mL) for 5 days. Next, cells were cultured in puromycin-free media for additional 72 h and the surviving, puro-resistant cells were plated as a single cell-derived clones to form colonies. After culturing, the colonies were validated for BLM KO by Western blotting and ultra-deep next generation sequencing. The two clones with the deletion of BLM fragments and a lowest expression of BLM protein were taken for further experiments.

### Cell viability and proliferation assays

Cell viability was determined using a MTT metabolism test as described [44]. Briefly, at given times after treatment, MTT stock solution was added to a final concentration of 0.5 mg/mL and after 1 h formazan crystals were dissolved in DMSO. Optical densities were measured at 570 nm and 620 nm using a scanning multiwell spectrophotometer. Cell proliferation was assessed using ELISA BrdU kit (Roche Diagnostics GmbH, Germany) according to the manufacturer’s protocol. For MTT and BrdU assays, cells were seeded at a density of 4 × 10^3^ cells/well.

### Cell cycle and cellular granularity analyses

For cell cycle and cellular granularity analysis, cells were seeded at a density of 2 × 10^5^ cells/well. Cell cycle analysis was performed by flow cytometry using BD Pharmingen PI/RNase Staining Buffer (BD Biosciences, USA). Briefly, cells were collected by trypsinization, fixed in 70% ethanol and stained in PI buffer (500 μL/1 × 10^6^ cells). DNA content analyses were performed using FACScalibur flow cytometer (BD Biosciences, USA) and the BD CellQuest Pro 6.0 software (BD Biosciences, USA). At least 10,000 events were analysed for each sample.

### Ectopic p53 expression and functional analyses

LN18 cells (2.5 × 10^5^ cells/well) were co-transfected with the plasmid carrying the gene encoding a wild-type p53 under the CMV promoter (pC53-SN3, 0.1 µg) [45] or empty vector (pcDNA3.1, 0.3 µg) and the plasmid encoding EGFP protein (pEGFP-N1, 0.3 µg) using Lipofectamine 2000 (Invitrogen, USA). Transfection efficiency and the p53 transcriptional activity were assessed with the p53-responsive luciferase reporter assay, based on two constructs: pPG13-Luc and pMG13-Luc, as described [24]. At 24 h after transfection cells were trypsinised, fixed and stained in 15 µM DRAQ5 in 4% PFA solution and a cell cycle was analysed using FACScalibur flow cytometer (BD Biosciences, USA) and the BD CellQuest Pro 6.0 software (BD Biosciences, USA). At least 10,000 events of GFP^+^ cells were analysed for each sample.

### RNA isolation, library preparation, sequencing and bioinformatic analysis

Total RNA was extracted from glioma cells using RNeasy Mini kit (Qiagen, Germany) and purified on Rneasy columns. Next, RNA was used to synthesise cDNA using SuperScript™ III Reverse Transcriptase (Invitrogen, USA). Quantity and quality of RNA was estimated using Agilent Bioanalyzer. Gene expression was evaluated by quantitative real-time PCR (qPCR) on Quantum Studio with 20 ng of cDNA in duplicates using TaqMan™ Fast Universal PCR Master Mix (ThermoFisher, USA) in 10 µL reaction with a listed set of primers (Supplementary Table S1). The amplified product was normalised to the endogenous expression of glyceraldehyde-3-phosphate dehydrogenase mRNA *(GAPDH)* and represented as delta Ct values.

Quality and integrity of RNA was assessed with Agilent 2100 Bioanalyzer using a RNA 6000 NanoKit (Agilent Biotechnologies, Santa Clara, CA, USA). PolyA enriched RNA libraries were prepared using the KAPA Stranded mRNA Sample Preparation Kit (Kapa Biosystems, Wilmington, MA, USA). Transcriptomic data were analysed as follows: fastq files were aligned to hg38 human reference genome with STAR program [46], and reads were counted to genes using feature Counts algorithm SUBREAD package [47]. Gene counts were normalised with the FPKM method, and differential analysis was performed using the DESeq2 [48]. Genes were considered to be differentially expressed (DE) with FDR corrected *p*-value < 0.05. Kyoto Encyclopedia of Genes and Genomes (KEGG) pathway analyses were performed using R package clusterProfiler [49] to annotate the functions of differentially expressed (DE) mRNAs. Data are available in NIH GEO database with the accession number GSE214931.

### Protein isolation and western blotting

Whole-cell protein extracts were prepared, resolved by electrophoresis and transferred to a nitrocellulose membrane as described [50]. After blocking with 5% non-fat milk in TBST (Tris-buffered solution pH 7.6, 0.01% Tween-20) the membranes were incubated overnight with primary antibodies: anti-BLM, anti-c-PARP, anti-p-c-CASP3, anti-c-CASP7 and anti-GAPDH diluted in a TBST with 3% bovine serum albumin (BSA) or 1 h with horseradish peroxidase (HP) – conjugated anti-β-actin antibody diluted in 5% non-fat milk in TBST. The primary antibody reaction was followed by 1 h incubation with HP-conjugated anti-rabbit IgG or anti-mouse IgG, which were diluted in TBST. Immunocomplexes were detected using an enhanced chemiluminescence detection system (ECL) and Chemidoc (Biorad). Membranes were stripped in 100 mM glycine and 20% SDS buffer, pH 3.0 for 30 min at RT, washed, blocked and re-probed with antibodies. Band intensities were measured by a densitometric analysis of immunoblots using the BioRad Image Lab (ver. 5.2) software. For antibody specifications, catalogue numbers, and dilutions, see Supplementary Table S1.

### Immunohistochemistry on tissue microarrays (TMA)

BLM expression across glioma samples was evaluated on glioma tissue microarray (US Biomax, Rockville, MD, USA). Paraffin-embedded tissue sections (5 μm) were incubated at 60 °C for 30 min and deparaffinized by incubation in xylene, followed by incubation in ethanol (100, 90, 70%), and rehydrated. Epitopes were retrieved by oven boiling in a pH 6.0 citrate buffer for 30 min. Endogenous peroxidase was blocked in 0.3% H_2_O_2_ in 10% methanol for 30 min followed by blocking with 10% horse serum. Sections were incubated overnight at 4 °C with the rabbit anti-BLM primary antibody. Next, specimens were washed in PBS, incubated with biotinylated horse immunoglobulin, then with HP-conjugated avidin for 60 min, and finally with 3,3′-diaminobenzidine (DAB). Sections were counterstained with hematoxylin (Sigma-Aldrich, Germany), washed, dehydrated and mounted. For reagent specifications, catalogue numbers, and concentrations, see Supplementary Table S1. Images were acquired with the Leica DM4000 B microscope operating with the Application Suite ver. 2.8.1 software (Leica Microsystems CMS, Switzerland). The scoring was performed by two independent scientists based on positive staining of the BLM protein in the nucleus or cytoplasm, and marked as '1' if staining was “present” or '0' if absent.

### Immunocytochemistry

Cells were seeded onto glass coverslip at a density of 3 × 10^5^ cells. After 24 h cells were fixed with 4% PFA pH 7.2, washed, permeabilized with 0.1% Triton-X100 and blocked in mix of 2% donkey serum and 1.5% foetal bovine serum, followed by 2 h incubation with rabbit anti-BLM and mouse anti-γH2AX, or mouse anti-γ-tubulin primary antibodies. Cells were then washed in PBS, incubated with donkey anti-rabbit Alexa Fluor A555 and donkey anti-mouse Alexa Fluor A488, or donkey anti-mouse Alexa Fluor A555, counterstained with DAPI and mounted. For reagent specifications, catalogue numbers, and concentrations, see Supplementary Table S1.

### Quantification of senescence-associated β-galactosidase-positive cells

The activity of senescence-associated β-galactosidase (SA-β-gal) was detected, as described [51]. Briefly, cells were seeded at density 2 × 10^4^ cells and after the treatment fixed with 2% formaldehyde and 0.2% glutaraldehyde in PBS, washed, and incubated overnight at 37 °C in the solution containing 1 mg/mL 5-bromo-4-chloro-3-indolyl-β-D-galactopyranoside, 5 mM potassium ferrocyanide, 5 mM potassium ferricyanide, 150 mM NaCl, 2 mM MgCl_2_, and 0.1 M phosphate buffer, pH 6.0. Cells were counted under a light Nikon Eclipse 50i microscope (Minato) and percentages of SA-β-gal-positive cells were calculated.

### Statistical analysis

All biological experiments were performed on 3–5 independent cell passages. Results are expressed as means ± standard deviation (SD). *P*-values were calculated using chi-square, two-tailed t test or one-way ANOVA followed by appropriate post-hoc test using GraphPad Prism v6 (GraphPad Software, USA). The precise description of statistics is given in figure captions. Differences were considered statistically significant for *p* values < 0.05. Moreover, we calculated the odds ratio (OR), and effect size (Hedge’s ‘g’ between the groups) [52]. We used Mantel–Haenszel Odds Ratio estimator, with repetitions as strata, with confidence interval CI95% based on Robins–Breslow–Greenland estimator of variance [53, 54]

## Supplementary information

Supplementary figure’s caption

Supplementary figure S1

Supplementary figure S2

Supplementary figure S3

supplementary table 1

## Data Availability

RNAseq data are available in NIH GEO database with the accession number GSE214931. All other data supporting the findings of this study are available within the paper or its supplementary information are available from the corresponding author upon reasonable request.
