# Supplementary material for: BLM helicase overexpressed in human gliomas contributes to diverse responses of human glioma cells to chemotherapy
Source: Cell Death Discov. 2023 May 11;9:157. doi: 10.1038/s41420-023-01451-9 (PMC10175545; doi:10.1038/s41420-023-01451-9)
Supplement: Supplementary file 1 — Supplementary figure’s caption [file 41420_2023_1451_MOESM1_ESM.docx]

**Figure S1. Gating strategy for flow cytometry analysis**

Events corresponding to cells were gated on SSC-A vs FSC-A plots. Events were further analyzed for the GFP^+^ and GFP^-^ cells stained with DRAQ5 dye. Histograms show the cell cycle phase with additional polyploidy state.

**Figure S2. Uncropped Western blots**

Uncropped western blots related to figure 3D, 3F of WT and BLM KO LN18 and LN229 cells.

**Figure S3. Cell morphology in control and stress conditions**

Cell morphology visualised by F-actin staining in control and after double, TMZ and OLA, treatment in **(A)** LN18 and **(B)** LN229 cells.

**Table S1 Antibodies, reagents and software**

Specifications, catalogue numbers and dilutions of reagents used for experiments.
