# Supplementary figures and images for: BLM helicase overexpressed in human gliomas contributes to diverse responses of human glioma cells to chemotherapy
Source: Cell Death Discov. 2023 May 11;9:157. doi: 10.1038/s41420-023-01451-9 (PMC10175545; doi:10.1038/s41420-023-01451-9)

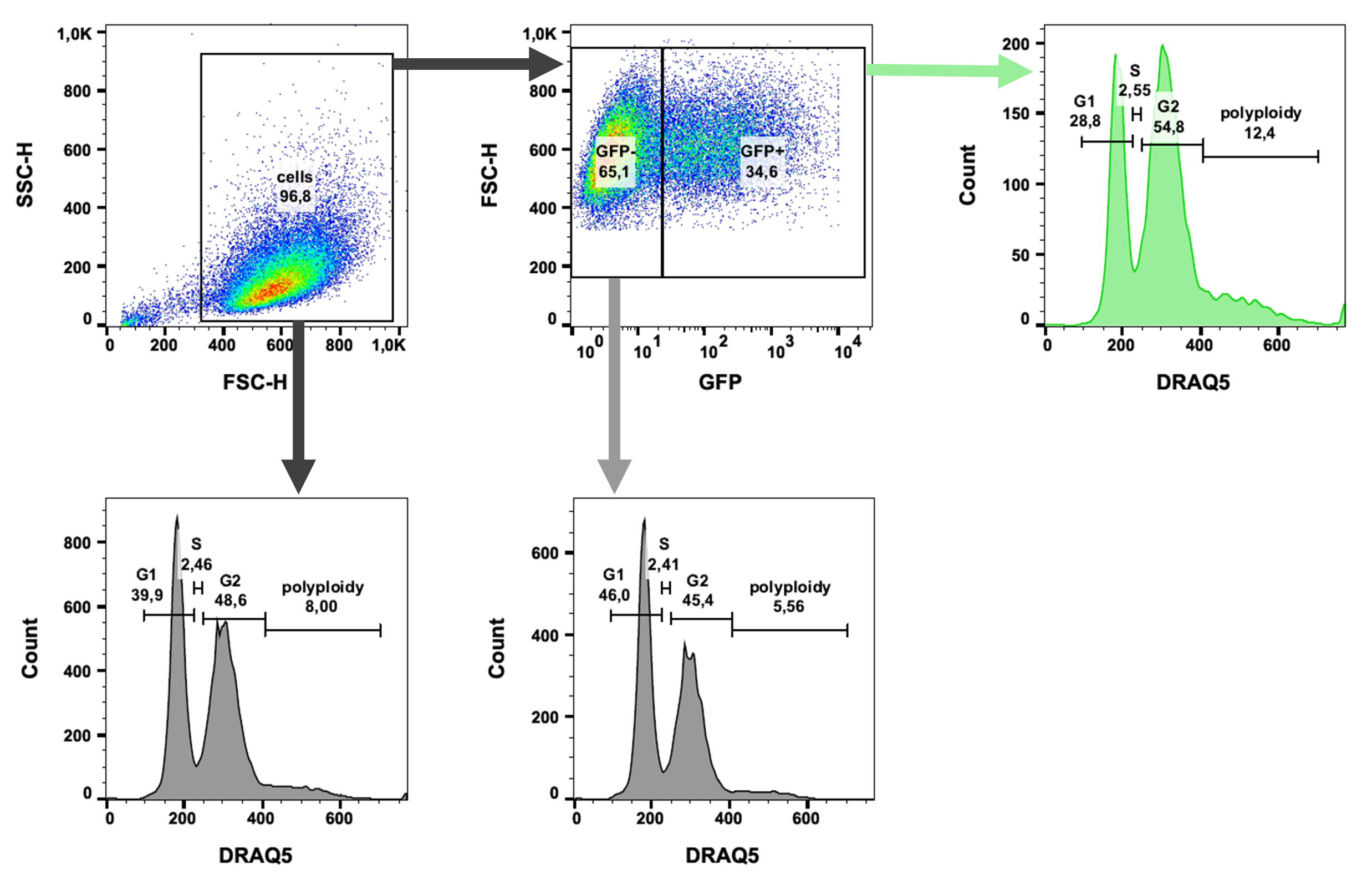

Supplement: Supplementary file 2 — Supplementary figure S1 [file 41420_2023_1451_MOESM2_ESM.tif]

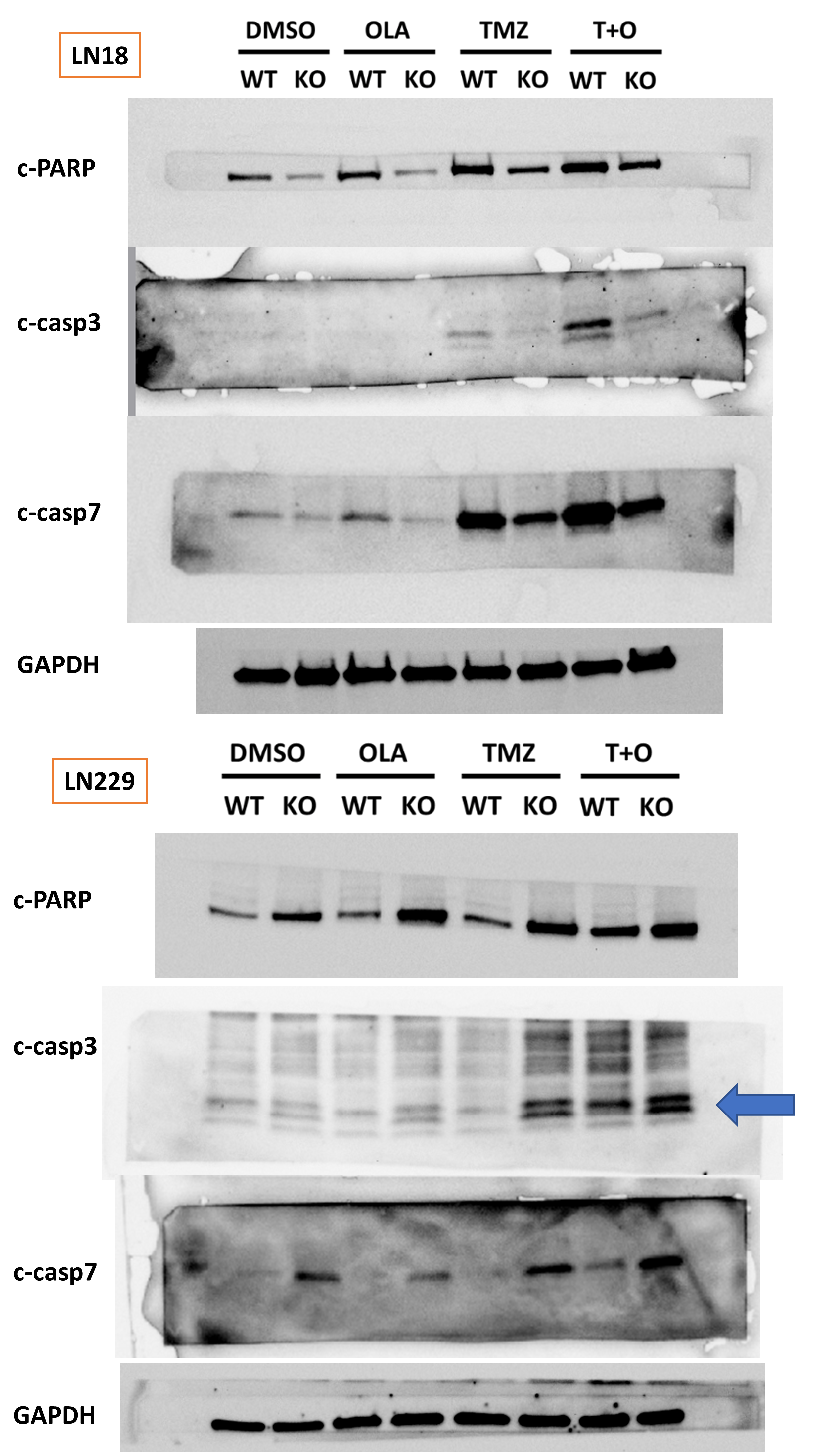

Supplement: Supplementary file 3 — Supplementary figure S2 [file 41420_2023_1451_MOESM3_ESM.tif]

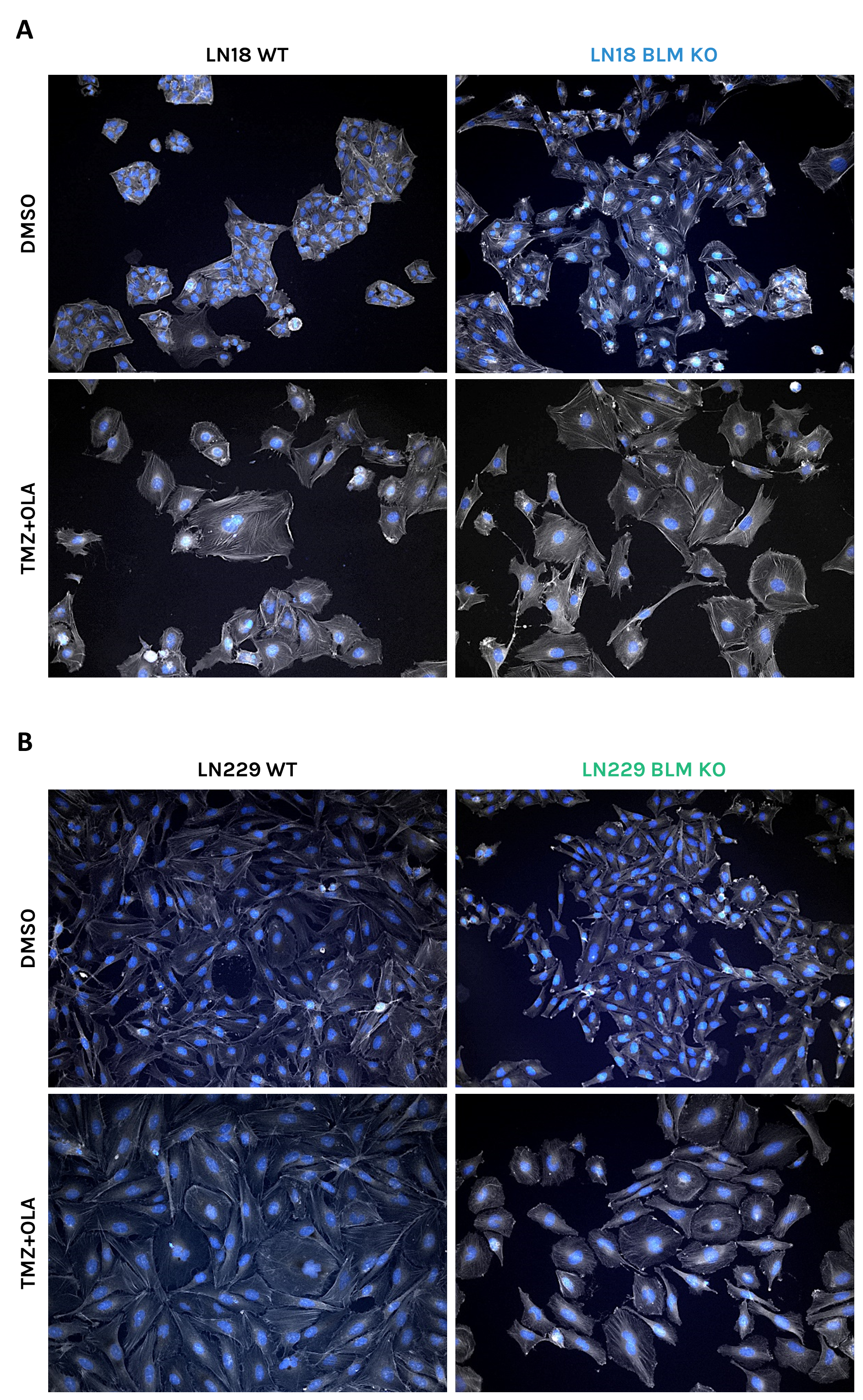

Supplement: Supplementary file 4 — Supplementary figure S3 [file 41420_2023_1451_MOESM4_ESM.tif]
