# Supplementary material for: BLM helicase overexpressed in human gliomas contributes to diverse responses of human glioma cells to chemotherapy
Source: Cell Death Discov. 2023 May 11;9:157. doi: 10.1038/s41420-023-01451-9 (PMC10175545; doi:10.1038/s41420-023-01451-9)
Supplement: Supplementary file 5 — supplementary table 1 [file 41420_2023_1451_MOESM5_ESM.docx]

| **Antibodies used for stainings** | | | | | |
| --- | --- | --- | --- | --- | --- |
| **Antibody** | **Clone** | **Manufacturer** | **Cat. number** | | **Dilution** |
| anti-BLM | - | Abcam | ab2179 | | 1:200 |
| anti-γtubulin | C-11 | SantaCruz | sc-17787 | | 1:100 |
| anti- γH2AX | 9F3 | Abcam | ab26350 | | 1:1000 |
| horseradish peroxidase-conjugated horse anti-mouse | - | Vector | BA2000 | | 1:200 |
| horseradish peroxidase-conjugated horse anti-rabbit | - | Vector | BA1100 | | 1:200 |
| donkey anti-rabbit A555 | - | ThermoFisher | A31572 | | 1:1000 |
| donkey anti-mouse A488 | - | ThermoFisher | A21202 | | 1:1000 |
| ExtrAvidin™−Peroxidase | - | Sigma-Aldrich | E2886 | | 1:200 |
| **Antibodies used for immunoblotting** | | | | | |
| anti-cleaved PARP | - | Cell signaling | 9541S | | 1:1000 |
| anti-cleaved Caspase3 | - | Cell signaling | 9661S | | 1:1000 |
| anti-cleaved Caspase7 | - | Cell signaling | 9491S | | 1:1000 |
| anti-GAPDH |  | Millipore | MAB374 | | 1:1000 |
| horseradish peroxidase-conjugated monoclonal anti-β-actin | AC-15 | Sigma Aldrich | A3854 | | 1:40000 |
| horseradish peroxidase-conjugated anti-rabbit IgG | - | Vector | PI-1000 | | 1:10000 |
| horseradish peroxidase-conjugated anti-mouse IgG | - | Vector | PI-2000 | | 1:10000 |
| **TaqMan™ primers and reagents** | | | | | |
| **Reagent** | | | | | **Source** |
| TaqMan™ Fast Universal PCR Master Mix (2X), (#4352042, lot:01029725) | | | | | ThermoFisher |
| human *BLM*: Hs00172060_m1 (lot: 1362591) | | | | | ThermoFisher |
| human *GAPDH*: Hs02758991_g1 (lot: 1853474) | | | | | ThermoFisher |
| **Other reagents** | | | | | |
| **Reagent** | | | | **Source** | |
| MVP Human Brain Total RNA (#540005, lot: 0006127195) | | | | Agilent | |
| Total RNA - Human Adult Normal Tissue (#R1234035-50, lot: B304105) | | | | Biochain | |
| Invitrogen™ Ambion™ FirstChoice™ Human Brain Reference RNA (#AM6050, lot: 1207015) | | | | Invitrogen | |
| Temozolomide | | | | Sigma-Aldrich | |
| Olaparib | | | | MedChemExpress | |
| 3-amino Benzamide | | | | Cayman Chemicals | |
| Rucaparib | | | | Cayman Chemicals | |
| Tioguanine | | | | Cayman Chemicals | |
| Etoposide | | | | Cayman Chemicals | |
| Cell Proliferation ELISA, BrdU colorimetric kit (# 11647229001) | | | | Roche | |
| BD Pharmingen PI/RNase Staining Buffer (# 550825 lot:7227966) | | | | BD Biosciences | |
| DRAQ5 (# 62251, lot:521DR50200) | | | | ThermoFisher | |
| CRISPRCLEAR™ Transfection Ready Kit #ASK-7010, LOT: CK036d  BLM.g1 GTTGGGTAGAGGTTCACTGA | | | | Applied Stem Cell | |
| **Software** | | | | | |
| GraphPad Prism 6.07 | | | | | |
| FlowJo 10 | | | | | |
| BD CellQuest Pro 6.0 | | | | | |
